# Deficits in episodic future thinking following acute alcohol consumption

**DOI:** 10.1007/s00213-022-06136-2

**Published:** 2022-04-13

**Authors:** Morgan Elliott, Gill Terrett, H. Valerie Curran, Natalie De Bono, Peter G. Rendell, Julie D. Henry

**Affiliations:** 1 School of Behavioural and Health Sciences, Australian Catholic University, Melbourne, VIC Australia; 2 Clinical Psychopharmacology Unit, University College London, London, UK; 3 School of Psychology, The University of Queensland, Brisbane, QLD Australia

**Keywords:** Acute alcohol consumption, Episodic future thinking, Neurocognitive function, Autobiographical interview, Sex differences

## Abstract

**Rationale:**

Acute alcohol consumption adversely affects many cognitive abilities, including episodic memory and executive functioning. However, no study to date has tested whether these acute effects of alcohol also extend to episodic future thinking (EFT). This is a surprising omission given that EFT refers to the ability to imagine oneself experiencing the future, a highly adaptive ability that has been implicated in many important functional behaviours. EFT is also thought to impose demands on episodic memory and executive control.

**Objectives:**

The current study was designed to provide the first test of whether a moderate dose of alcohol influences EFT and whether any observed EFT difficulties are secondary to broader problems in episodic memory and executive functioning. Sex differences in EFT following acute alcohol consumption were also examined.

**Methods:**

One hundred and twenty-four healthy adult social drinkers were recruited and randomly assigned to either the alcohol (*n* = 61) or placebo (*n* = 63) condition. Participants were administered a dose of 0.6 g/kg alcohol or a matched placebo drink.

**Results:**

Relative to the placebo condition, EFT was impaired by acute alcohol consumption. This impairment was underpinned by broader difficulties with episodic memory, but not executive functioning. There were no sex differences in EFT performance following acute alcohol use.

**Conclusion:**

These data provide novel insights into the effects of acute alcohol consumption on EFT and the broader cognitive mechanisms that contribute to these difficulties. The results are discussed in relation to their implications for understanding many of the maladaptive behaviours commonly associated with acute alcohol use.

Alcohol is one of the most commonly used substances worldwide, largely consumed for its relaxant and euphoric properties (Oscar-Berman and Marinković 2007). Alcohol contains ethanol, a central nervous system depressant associated with changes in subjective mood, psychomotor performance, and cognitive functioning (Eckardt et al. 1998; Fillmore 2007; Holloway 1994; Hull and Bond 1986; Weissenborn and Duka 2003). Acutely, alcohol exerts its effects by altering the physical properties of neural membranes and disrupting synaptic transmission (Charness 1990).

In terms of the effect of acute alcohol consumption on cognition, memory and executive function are two key abilities that have attracted particular attention in this literature. Consistent with neurobiological evidence showing that the brain regions impacted by acute alcohol consumption include areas strongly linked to memory function, such as the hippocampus (Jacob and Wang 2020; Squire et al. 2004), acute alcohol use has been associated with memory impairment (Curran and Weingartner 2002; Mintzer 2007; Ryback 1971; White 2003). However, although many of the key brain regions that support executive functions are also impacted by acute alcohol consumption (Oscar-Berman and Marinković 2007; Van Skike et al. 2019), the executive function literature is more mixed, and it has been suggested that any relationship may be dose and/or task dependent (for a review, see Day et al., 2015).

Another important aspect of cognition that may be affected by acute alcohol use, but which has not yet been tested empirically, is episodic future thinking (EFT). EFT refers to the ability to imagine or simulate a personal hypothetical future scenario (Atance and O'Neill 2001; Schacter et al. 2008; Szpunar 2010). It is therefore a highly adaptive ability that allows an individual to carefully plan their behaviour by pre-experiencing a future personal event and imagining the consequences of their actions, thus enhancing the likelihood of achieving desired outcomes, and avoiding undesirable ones (Suddendorf and Corballis 2007). Because deficits in EFT are linked to poor decision making, they have potentially profound consequences for daily functioning and independent living (Schacter et al. 2008, 2017; Szpunar 2010). Indeed, research has identified EFT deficits in many clinical groups that exhibit functional difficulties (D'Argembeau et al. 2008; Lind and Bowler 2010; Mercuri et al. 2015, 2018), and failures in EFT may potentially help to explain why acute alcohol consumption is linked to risk taking behaviours, such as unprotected sex, aggression and gambling (Field et al. 2010).

EFT difficulties in the context of acute alcohol consumption might be anticipated because of alcohol’s broader effects on episodic memory. Episodic memory refers to the ability to recall past personal experiences. According to the *constructive episodic simulation hypothesis*, humans typically extract, recombine and integrate the recollection of such past experiences in the construction of novel future events (Addis 2018; Roberts et al. 2018; Schacter and Addis 2007b). That is, episodic memory provides the foundation from which the future is imagined (Suddendorf et al. 2009). EFT and episodic memory also engage a similar core brain network (Addis et al. 2008; Schacter et al. 2017), suggesting that these brain regions function adaptively to simulate possible future events by integrating information from past experiences (Schacter et al. 2007). Additional support for the claim that episodic memory is an important contributor to EFT comes from studies that have shown EFT and episodic memory to be significantly correlated in both healthy controls and clinical populations (see Schacter and Addis 2007a for a review), and there is evidence that poorer EFT may at least partially reflect poorer episodic memory (e.g. Mercuri et al. 2015, 2018). Therefore, problems with EFT may be expected to be a corollary of any effect of acute alcohol consumption on episodic memory.

Problems with EFT may also be anticipated as a secondary consequence of acute alcohol-related executive function difficulties. As noted, EFT involves simulating a future scenario in imagination, a constructive process that requires an individual to flexibly recombine past memories into coherent, novel future events (Schacter and Addis 2007b; Suddendorf and Henry 2013). As such, this process has been hypothesised to involve cognitive flexibility (an aspect of executive function) to not only disengage from the present moment to focus on the imagined future, but to also reformulate past experiences to simulate that future event. It has also been suggested that EFT requires inhibitory control to impede irrelevant information and working memory to mentally manipulate information (Schacter and Addis 2007b; Suddendorf 2010; Suddendorf and Corballis 2007). However, empirical support for a relationship between EFT and executive function is mixed, with some studies identifying significant associations (e.g. Cole et al. 2013; Mercuri et al. 2018), whilst others have failed to identify any relationship (e.g. Gott and Lah 2014; Mercuri et al. 2015). Additionally, as previously noted, findings in relation to the effect of acute alcohol use on executive functions have been similarly mixed. As a consequence, the extent to which problems with EFT may be expected to arise due to executive dysfunction is unclear.

The primary aim of the present study was therefore to provide the first empirical test of whether EFT performance is impaired following a moderate dose of alcohol (0.6 g/kg). The second aim was to examine whether any observed EFT impairments were related to acute alcohol-induced deficits in episodic memory and/or executive function. The key predictions were (i) relative to a placebo condition, participants in the alcohol condition would perform significantly worse on EFT and episodic memory. It was also anticipated that (ii) episodic memory would significantly contribute to EFT performance in both conditions. In light of the mixed findings reported previously, less clear was whether executive functioning would be impaired by alcohol and contribute to EFT impairment. The final, exploratory aim of this study was to examine sex differences in EFT in the context of acute alcohol consumption. This question was of interest because there is some evidence to suggest that there are sex differences in the pharmacokinetics of acute alcohol use and its resulting impact on cognitive functioning (Erol and Karpyak 2015; Fama et al. 2020; Mumenthaler et al. 1999).

## Methods

### Participants and design

One hundred and twenty-four healthy adult social drinkers (62 men) aged 18–37 (M = 24.35, SD = 4.16) were recruited via community advertisements and social networks. Social drinking^1^ was defined as the average weekly consumption of between 2 and 25 standard units of alcohol for women and 2–36 standard units of alcohol for men (Griffiths et al., 2012). Exclusion criteria were previous or current neurological condition or major psychiatric illness; history of alcohol or other substance dependence; the use of prescription medication that required abstinence from alcohol. Participants were also excluded if English was not their first language. Participants were asked to refrain from using alcohol or illicit drugs in the 24 h prior to testing and reminded of this via text message at least a day in advance of the testing session. Participants confirmed abstinence via self-report and a BrAC measurement of zero prior to commencing. All participants were reimbursed AU$60 for their time.

Participants were randomly assigned to either the alcohol (*n* = 61; 30 men) or placebo (*n* = 63; 32 men) condition in a double-blind, independent group design. The groups did not differ in sex composition, $\chi$ ^2^ (1124) = 0.03, *p* = 0.86. As shown in Table 1, the groups also did not differ in age, years of education, premorbid intelligence as measured by the *Spot the Word* test (Baddeley et al. 1993), and negative affect, as measured by the *Hospital Anxiety and Depression Scale* (Zigmond and Snaith 1983).

**Table 1:** Participant characteristics

	Alcohol condition *n* = 61	Placebo condition *n* = 63			
	*M* (*SD*)	*M* (*SD*)	*t* (122)	*d*	*p*
Age (in years)	24.25 (4.17)	24.46 (4.19)	0.29	.05	.78
Years of education	16.13 (2.24)	16.21 (2.08)	0.21	.04	.83
Premorbid IQ	47.00 (4.27)	46.87 (4.46)	0.16	.03	.87
Mental health					
Depression	3.03 (2.56)	2.33 (2.28)	1.61	.29	.11
Anxiety	7.07 (3.64)	7.03 (4.10)	0.05	.01	.96

Premorbid IQ as measured by the *Spot the Word* test

Table 2 presents descriptive and inferential statistics for alcohol use characteristics (total sample, males and females), and independent samples *t*-tests comparing total sample group differences (alcohol vs. placebo) for each of the alcohol use variables. The alcohol and placebo groups did not differ in age of first alcoholic drink, average quantity of alcohol consumed per week in standard units, speed of drinking, number of times ‘drunk’ within the past 6 months and percentage of times drinking until drunk, in addition to *Alcohol Use Questionnaire* (*AUQ*; Mehrabian and Russell 1978) outcome scores.

**Table 2:** Alcohol use demographics (as measured by the Alcohol Use Questionnaire) for the total sample and separately for males and females

		Alcohol condition *n* = 61		Placebo condition *n* = 63				
		*M*	*SD*	*M*	*SD*	*t* (122)^a^	*d*	*p*
Age of first alcoholic drink	Total sample	15.18	2.03	15.13	1.85	0.15	.03	.88
	Males	14.80	2.24	15.28	1.99			
	Females	15.55	1.77	14.97	1.72			
Alcohol units per week	Total sample	8.21	5.30	7.76	5.75	0.45	.08	.65
	Males	9.92	5.73	8.66	6.14			
	Females	6.56	4.32	6.84	5.25			
Alcohol units per hour	Total sample	2.07	0.10	1.90	0.78	0.85	.15	.40
	Males	2.47	1.17	1.91	0.69			
	Females	1.63	0.56	1.90	0.88			
Number of times drunk^b^	Total sample	12.05	13.39	10.95	17.65	0.39	.07	.70
	Males	16.98	16.09	15.41	22.21			
	Females	7.27	7.78	6.35	9.56			
Percentage of times drinking until drunk^b^	Total sample	31.98	27.87	25.11	26.53	1.41	.25	.16
	Males	37.78	28.49	31.70	31.05			
	Females	26.35	26.50	18.31	19.09			
*AUQ* score	Total sample	34.82	21.93	31.32	26.24	0.81	.15	.42
	Males	44.33	24.27	38.03	31.64			
	Females	25.63	14.66	24.39	17.04			
*AUQ* binge score	Total sample	26.61	18.85	23.59	22.61	0.81	.15	.42
	Males	34.41	21.61	29.37	27.66			
	Females	19.06	11.83	17.63	13.90			

^a^Independent samples *t*-tests comparing alcohol and placebo conditions total sample means

^b^ ‘Drunk’ is defined as the loss of coordination, nausea, and/or inability to speak clearly

### Alcohol administration

A research assistant was responsible for the mixing of drinks and BrAC measurements to ensure that the researcher remained blind to condition (alcohol or placebo). For participants assigned to the alcohol condition, alcohol was administered at a dose of 0.6 g/kg of body weight. This dose was chosen because it is considered to be a good representation of moderate alcohol intoxication and is commonly experienced by social drinkers without considering themselves to be intoxicated or cognitively impaired to a notable degree. Closely following the procedure outlined by Leitz et al. (2009), a total of 500 ml of liquid was prepared containing 96% ethanol diluted with tonic water and lime cordial, and divided equally into 10 cups containing 50 ml portions. Lime cordial was used to mask the taste of alcohol. For participants in the placebo condition, the placebo beverage consisted of 500 ml of liquid divided equally into 10 cups containing 50 ml portions of tonic water and lime cordial only. Participants in both conditions were provided with their respective 10 beverages and were required to consume the drinks at 3-min time intervals in the presence of the researcher.

To maintain the level of alcohol in the blood over the entire testing period of up to 180 min, participants in the alcohol condition were given two sets of top-up drinks, each containing 0.1 g/kg dose of alcohol, tonic water and lime cordial, whilst those assigned to the placebo condition were given two sets of placebo drinks. Each top-up drink contained two 50 ml portions which were administered at approximately 80- and 120-min into the testing session. Previous research has shown that a dose of 0.1 g/kg alcohol administered to participants in the alcohol condition as top-up drinks can be used to maintain a steady BrAC over the entire testing period (Leitz et al. 2009; Paraskevaides et al. 2010). Each participant completed four BrAC measurements administered by the research assistant throughout the testing session using a Lion Alcolmeter 700 breathalyser. Participants were breathalysed at least 20 min after drink administration to ensure that the BrAC reading for participants in the alcohol condition was not affected by residual alcohol within the mouth, and at the conclusion of the tasks to avoid distraction from task completion and adversely impacting performance.

### Procedure

Testing took place in one session of up to 180 min duration, with breaks taken as needed. After completing the baseline BrAC measurement, participants were weighed to allow the appropriate dose of alcohol to be calculated for those allocated to the alcohol condition. Participants then completed the tasks detailed in Table 3. The study was approved by the Australian Catholic University Human Research Ethics Committee and all participants provided informed consent.

**Table 3:** Procedure: tasks performed with approximate corresponding times (min), including average blood alcohol concentration (BrAC) of participants assigned to the alcohol condition (total sample and separately for males and females)

Time	Tasks and measures	Alcohol group BAC
0 min	Informed consent Baseline BrAC Weigh participant *Spot the word* Initial drink administration (0.6 g/kg alcohol OR placebo) Background questionnaire *Hospital Anxiety and Depression Scale* *Alcohol Usage Questionnaire*	Total sample M = 0.000
45 min	End of drink administration *Verbal Fluency Task* *Hayling Sentence Completion Test* *Trail Making Test*	
75 min	BrAC time 2 reading Top up drink (0.1 g/kg alcohol OR placebo)	Total M = 0.064, SD = 0.014 Males M = 0.061 SD = 0.016 Females M = 0.067, SD = 0.011
120 min	BrAC time 3 reading Top up drink (0.1 g/kg alcohol OR placebo) *Autobiographical interview*	Total M = 0.075, SD = 0.013 Males M = 0.070 SD = 0.014 Females M = 0.079, SD = 0.011
170 min	BrAC time 4 reading Debriefing, guess condition and payment	Total M = 0.074, SD = 0.014 Males M = 0.068 SD = 0.013 Females M = 0.080, SD = 0.013

### Measures

#### Episodic future thinking and episodic memory

The *Adapted Autobiographical Interview* (*AI;* Addis et al., 2008) is a semi-structured interview that was used to provide a measure of EFT and episodic memory. In this task, participants are provided with a set of six cue words and asked to describe an event relating to the cue word in as much detail as possible as if they were actually experiencing the event. The event had to be either one that they had previously experienced in real life (past condition) or a novel future event (future condition). Cue words were selected from the ‘*Affective Norms for English Words*’ list (*ANEW*; Bradley and Lang 1999), and included two positive words (*birthday, vacation*), two neutral words (*bench, taxi*), and two negative words (*nightmare, accident*; valence ratings M = 8.0, M = 4.8, M = 2.0, respectively). Three cue words, one from each valence, were administered for each temporal direction (i.e. past and future). To reduce cognitive load, and facilitate participants’ understanding and adherence to instructions, all three cue words were administered for one temporal direction before commencing the other temporal direction. The presentation of temporal direction and cue words was counterbalanced across participants, leading to six versions of the task. Administration, training of scorers, and scoring procedures closely followed Addis et al. (2008).

##### AI procedure

One interviewer was trained in the administration of the *AI* and conducted all interviews. Participants were provided with the instructions and were given a demonstration prior to the administration of test cues. For each trial, participants were instructed to generate as many internal details as possible (i.e. episodic information specific to the central event, such as thoughts, feelings and sensory information) about an event in relation to each cue word (e.g. *birthday*). Participants were given 3 min to describe this event which had to be either one that they could imagine themselves experiencing within the next 3 years (future condition instruction) or one that they had experienced within the past 3 years (past condition instruction). For future events, participants were instructed that the event they described could be creative, but not totally unrealistic. Additionally, participants were directed to describe the event from their own subjective perspective and told that the event should be less than 1 day in duration and refer to a specific place and time. When necessary, set prompts were provided to clarify instructions and encourage further description of the event, such as, ‘Is there anything else you can tell me?’ and ‘Are there any other details that come to mind?’ Responses were recorded using a digital audio recorder and transcribed for scoring.

##### AI scoring

For each trial, a central event was identified, and then details segmented and categorised as either *internal* (episodic information specific to the central event) or *external* (non-episodic information, including repetitions, semantic information and information not specific to the central event). The total number of internal details generated across the three future trials was the primary measure of EFT, and the total number of internal details generated across the three past trials was the primary measure of episodic memory. The total number of external details generated across the three future and three past trials represents error. Three independent scorers blind to the project aims and participant condition scored the transcripts; Cronbach’s alphas were 0.98 for internal and 0.92 for external details.

#### Executive functions

##### Cognitive initiation

The *Verbal Fluency Task* was used to provide an index of the cognitive initiation component of executive control (Strauss et al. 2006). Two variants were used: phonemic fluency, which requires generation of as many words as possible on the basis of orthographic criteria (in the present study, F, A and S), and semantic fluency which requires generation of words according to categorical criteria (in the current study, types of animals). A total verbal fluency score was calculated by summing the number of correct responses for phonemic and semantic fluency.

##### Inhibitory control

The *Hayling Sentence Completion Test* provided a measure of the inhibitory control component of executive control, and was administered and scored according to Burgess and Shallice (1997). The task contains two parts: Part A, in which participants are required to sensibly complete 15 sentences (e.g. “He posted a letter without a … [*STAMP*]”); and Part B, in which participants are required to complete an additional 15 sentences, but with unrelated words (e.g. “Her new shoes were the wrong… [*PENCIL*]”). Both parts are timed and recorded in seconds, and performance is calculated by tallying the number of errors and the total time taken to complete both parts of the test, which is then converted to a scaled score.

##### Cognitive flexibility

The *Trail Making Test* (*TMT*) contains two parts that are timed and recorded in seconds, and was used to provide a measure of the cognitive flexibility component of executive control (Arbuthnott and Frank 2000; Tombaugh et al. 1999). The *TMT* was administered according to standardised guidelines (Strauss et al. 2006). In Part A, participants are required to draw one continuous line to connect the numbers 1 to 25 in numerical order, and therefore provides a baseline measure of psychomotor speed. In Part B, participants are required to draw one continuous line to connect numbers and letters in sequential increasing and alternating order (i.e. 1-A-2-B-3-C, etc.). The executive score is calculated by subtracting the time taken for Part A from Part B, with lower scores reflecting better executive control.

#### Pre-morbid intelligence

Premorbid intelligence was assessed using the validated test of word-recognition, the *Spot the Word* task (Baddeley et al. 1993). In this task, participants are provided with 60 pairs of items. Each pair of items contains a real word and an invented word constructed to resemble a real word but has no meaning. Participants are required to identify and circle which item is the real word in each pair of items. The *Spot the Word* task is strongly correlated (*r* = 0.83) with the *National Adult Reading Test* (Baddeley et al. 1993), a well-established measure of premorbid intelligence quotient (IQ).

#### Alcohol use

The *AUQ* (Mehrabian and Russell 1978) is a 12-item self-report questionnaire that provides a reliable measure of alcohol drinking habits within the past 6 months (Townshend and Duka 2002). The *AUQ* score is calculated based on the total number of alcohol units per week, speed of drinking, number of times being drunk and percentage of times getting drunk whilst drinking alcohol. The *AUQ* was also used to provide a binge drinking score, which was calculated using the Townshend and Duka (2002) *AUQ* binge score equation.

### Statistical analysis

All statistical tests were two-tailed and conducted using IBM SPSS Statistics, version 26.0 (IBM Corp). An alpha level of *p* < 0.05 was considered significant, and effect sizes were estimated either using partial eta squared ($\eta$ ^2^_p_), Cohen’s *d*, or Pearson *r* correlations, depending on the analytic approach. Cohen (1988) defines *η*^2^_*p*_ effect sizes of 0.01 as small, 0.059 as medium and 0.138 as large, *d* effect sizes of 0.2 as small, 0.5 as medium and 0.8 as large, and *r* effect sizes of 0.1 as small, 0.3 as medium and 0.5 as large. There were no missing values; however, one case was identified as a univariate outlier in the *Verbal Fluency Task*, as were two cases in the *TMT*, with z-scores of more than 3.29 (Tabachnick and Fidell 2014). These outliers were replaced with scores $\pm$ 3SDs of the mean following Tabachnick and Fidell (2014).

## Results

### Blood alcohol concentration

The mean (SD) BrAC at baseline, 75-, 120- and 170-min testing are reported in Table 3, separately for males and females assigned to the alcohol group. Independent samples *t*-tests were conducted to compare sex differences in BrAC across the four BrAC measurements. All participants obtained a baseline BrAC of zero. Males and females obtained a similar BrAC on the second BrAC measurement, *t* (59) = 1.67, *p* = 0.10, *d* = 0.44. However, females obtained a significantly higher BrAC level than males on the third, *t* (59) = 2.92, *p* = 0.005, *d* = 0.71, and fourth BrAC measurements, *t* (59) = 3.68, *p* = 0.001, *d* = 0.92.

### Episodic future thinking and episodic memory

**Fig. 1:**
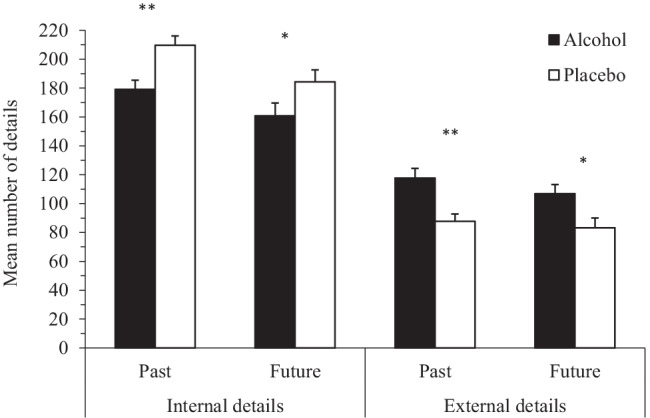
Mean number of internal and external details generated on the autobiographical interview as a function of condition (Alcohol, *n* = 61; Placebo, *n* = 63) and temporal direction (Past; Future). Error bars represent mean standard error

The number of details generated for past and future events is displayed in Fig. 1 as a function of *condition* (alcohol, placebo), *temporal direction* (past, future), and *detail type* (internal, external). As shown in Fig. 1, and confirmed by the formal analysis below, participants in the alcohol condition produced less internal details and more external details than participants in the placebo condition in both past and future conditions. A mixed 2 $\times$ 2 $\times$ 2 $\times$ 2 ANOVA was conducted with the between-groups variables, *condition* and *sex*, and the within-groups variables, *temporal direction* and *detail type*. There was no four-way interaction, *F* (1, 120) = 0.68, *p* = 0.41, *η*^2^_*p*_ < 0.01, and each three-way interaction was not significant: condition, temporal direction and detail type, *F* (1, 120) = 0.56, *p* = 0.46, *η*^2^_*p*_ < 0.01; sex, temporal direction and condition, *F* (1, 120) = 1.34, *p* = 0.25, *η*^2^_*p*_ = 0.01; sex, detail type and condition, *F* (1, 120) = 0.13, *p* = 0.72, *η*^2^_*p*_ < 0.01 and sex, temporal direction and detail type, *F* (1, 120) = 0.07, *p* = 0.80, *η*^2^_*p*_ < 0.01.

There was a two-way interaction between detail type and condition, *F* (1, 120) = 15.66, *p* < 0.001, *η*^2^_*p*_ = 0.12, but the other two-way interactions were not significant: temporal direction and condition, *F* (1, 120) < 0.01, *p* = 0.95, *η*^2^_*p*_ < 0.001; temporal direction and detail type, *F* (1, 120) = 2.40, *p* = 0.12, *η*^2^_*p*_ = 0.02; sex and condition, *F* (1, 120) = 0.55, *p* = 0.46, *η*^2^_*p*_ < 0.01; sex and temporal direction, *F* (1, 120) = 0.07, *p* = 0.79, *η*^2^_*p*_ < 0.01 and sex and detail type, *F* (1, 120) = 0.64, *p* = 0.43, *η*^2^_*p*_ < 0.01. Thus, sex did not interact with any other variable, and sex was also not a main effect: *F* (1, 120) = 0.22, *p* = 0.64, *η*^2^_*p*_ < 0.01. Temporal direction was also not an interaction effect, but was a main effect: *F* (1, 120) = 47.95, *p* < 0.001, *η*^2^_*p*_ = 0.29, indicating that collapsed across conditions, participants provided more details in the past (M = 148.6, SD = 28.7) than in the future condition (M = 133.87, SD = 33.37).

The significant interaction between detail type and condition was formally followed up with tests of simple effects. This interaction revealed that participants in the placebo condition produced more internal details (M = 197.00, SD = 50.99) than participants in the alcohol condition (M = 170.21, SD = 51.82), *F* (1, 120) = 8.29, *p* = 0.005, *η*^2^_*p*_ = 0.07. Conversely participants in the alcohol condition produced more external details (M = 112.26, SD = 42.31) than participants in the placebo condition (M = 85.55, SD = 42.31), *F* (1, 120) = 12.35, *p* = 0.001, *η*^2^_*p*_ = 0.09.

Finally, an independent samples *t*-test was conducted to examine if the alcohol and placebo conditions differed in the total amount of verbal output produced. The results revealed that there was no difference (*p* = 0.99) between the alcohol (M = 564.72, SD = 122.46) and placebo (M = 565.06, SD = 105.18) conditions in the total number of details generated across all six interview conditions.

### Executive functions

Descriptive and inferential statistics for the executive function measures are reported in Table 4. There were no differences between the alcohol and placebo conditions on any of the executive function measures assessed.

**Table 4:** Differential and inferential statistics for the executive function measures for participants in the alcohol and placebo conditions

	Alcohol condition *n* = 61	Placebo condition *n* = 63			
	*M* (*SD*)	*M* (*SD*)	*t* (122)	*d*	*p*
Executive function					
Cognitive initiation	58.46 (12.11)	61.32 (13.11)	1.26	0.23	.21
Inhibitory control	6.15 (1.00)	6.35 (0.88)	1.93	0.21	.24
Cognitive flexibility	40.65 (25.65)	34.29 (17.90)	1.61	0.29	.11

### Cognitive correlates of episodic future thinking

To investigate the cognitive correlates of EFT, Pearson product-moment correlations were calculated between EFT (indexed by future internal details), episodic memory (indexed by past internal details), and executive functioning, separately for each condition (i.e. alcohol, placebo). As shown in Table 5, significant correlations were found between EFT and episodic memory in both conditions, with better episodic memory performance associated with better EFT. However, for executive functioning, associations were inconsistent: EFT was positively correlated with only inhibitory control in the alcohol condition, and only cognitive initiation in the placebo condition.

**Table 5:** Correlations between episodic future thinking and measures of episodic memory and executive function, separately for the alcohol and placebo conditions

	Episodic future thinking	
	Alcohol condition *n* = 61	Placebo condition* n* = 63
Premorbid IQ	− .10	.16
Episodic memory	.45**	.60**
Executive function		
Cognitive initiation	.14	.30*
Inhibitory control	.29*	.03
Cognitive flexibility	− .19	.04

^*^p < 0.05, **p < 0.01

Premorbid IQ as measured by the *Spot the Word* test

### Regression analyses

To test whether the associations identified in the correlational analyses reflected unique contributions to EFT, hierarchical multiple regression analyses were conducted separately for the alcohol and placebo conditions (see Table 6). Premorbid IQ (as estimated by the *Spot-the-Word* Test) was entered in Step 1 in the analyses, episodic memory (past internal details) in Step 2, and executive functions in Step 3. The results suggest that the variables in the overall model predicted significant variance in performance in both the alcohol (total $R$ ^2^ = 0.25, *F* (5, 55) = 3.68, *p* = 0.006) and placebo condition (total $R$ ^2^ = 0.38, *F* (5, 57) = 6.88, *p* < 0.001). However, episodic memory was the only variable that significantly contributed to EFT in the alcohol and placebo condition in the final model.

**Table 6:** Hierarchical multiple regression analyses predicting episodic future thinking from premorbid IQ, episodic memory and executive functions, separately for the alcohol and placebo conditions

	Episodic future thinking					
	Alcohol condition *n* = 61			Placebo condition *n* = 63		
Predictor	$\Delta R$ ^2^	*B* (*SE*)	$\beta$	$\Delta R$ ^2^	*B* (*SE*)	$\beta$
Step 1	.01			.02		
Premorbid IQ		− 1.61 (2.09)	− .10		2.32 (1.88)	.16
Step 2	.19**			.34**		
Premorbid IQ		− 0.73 (1.91)	− .05		0.27 (1.58)	.02
Episodic memory		0.62 (0.17)	.44**		0.77 (0.14)	.60**
Step 3	.05			.02		
Premorbid IQ		− 1.14 (2.00)	− .07		0.25 (1.75)	.02
Episodic memory		0.56 (0.17)	.40*		0.77 (0.15)	.59**
Inhibitory control		10.97 (9.73)	.16		− 3.95 (8.48)	− .05
Cognitive flexibility		− 0.19 (0.38)	− .07		0.42 (0.40)	.11
Cognitive initiation		0.20 (0.75)	.04		0.27 (0.69)	.05
Total $R$ ^2^	.25*			.38**		

^*^p < 0.01, **p < 0.001

Premorbid IQ as measured by the *Spot the Word* test

## Discussion

These data provide the first empirical assessment of the capacity for EFT following the administration of a moderate dose of alcohol, offering further novel insight into the neurocognitive effects of acute alcohol consumption. Consistent with predictions, the results showed that, relative to the placebo condition, EFT was disrupted by a moderate level of alcohol consumption, with significantly fewer episodic details generated by participants in the alcohol condition when asked to imagine and describe a novel future scenario. These data supplement and extend prior research showing acute alcohol-related impairment in other aspects of cognitive functioning (e.g. Day et al. 2015; White 2003) by demonstrating that the capacity to imagine the self in the future is also adversely affected.

It is also important to note that the production of fewer episodic details in the future did not simply reflect a generally lower level of overall verbal output. The alcohol and placebo conditions did not differ in the total amount of details produced when describing future events, but rather, only differed on the types of details generated (i.e. internal vs. external). Specifically, the alcohol condition produced more non-episodic, external details than the placebo condition, despite explicit instructions given in the administration of the *AI* procedure to generate episodic content. As previously noted, overproduction of non-episodic details is considered indicative of error (Irish et al. 2011; Mercuri et al. 2015).

The identification of significant deficits in EFT following levels of acute alcohol consumption that would not be considered particularly high in modern society has potentially important practical implications. As noted earlier, EFT is important for daily functioning, decision making and problem solving, allowing humans to assess potential dangers and carefully plan any actions before performing them, thereby reducing the risk of harm (Suddendorf and Corballis 2007). Because EFT also plays a role in goal-oriented behaviour and emotion regulation, any difficulties engaging in EFT has the potential to cause motivational problems and irrational behaviour. In the context of substance use, problems with EFT may therefore present as increased risk-taking, maladaptive decision making, and a tendency to prioritise current needs over future goals that may potentially be more beneficial (Grant et al. 2000; Suddendorf and Corballis 2007). Indeed, deleterious behaviours commonly associated with alcohol intoxication include an increase in sexual risk taking, aggressive behaviour and drink driving, all of which may be caused and/or potentially reinforced by a reduced capacity for EFT. In addition, many therapeutic techniques for the treatment of alcohol use disorder require some degree of future thought, such as goal setting and the weighing up of future consequences. As a consequence, difficulties with EFT could potentially jeopardise treatment progress and may present an additional target for treatment. The current findings therefore have potentially important implications for the treatment of individuals with an alcohol use disorder.

### The potential role of other acute alcohol-related cognitive difficulties

The second key result to emerge was that the acute alcohol-induced problems with EFT appear to at least partially reflect broader problems with episodic memory. As predicted, and aligning with considerable prior literature, participants in the alcohol condition generated significantly fewer episodic details when asked to describe a personal past event relative to those in the placebo condition, indicating a deleterious effect of acute alcohol consumption on episodic memory. Additionally, a significant correlation was identified between episodic memory and EFT, with regression analyses showing that episodic memory was the only cognitive variable to significantly contribute to EFT (with this effect evident in both the placebo and alcohol conditions). These results therefore provide evidence that the negative effects of acute alcohol consumption on episodic memory contributes to poorer EFT. Thus, although they cannot directly speak to causality, they are consistent with the *constructive episodic simulation hypothesis*, which regards episodic memories as the basic materials from which hypothetical future scenarios are built (Addis et al. 2008; Buckner and Carroll 2007; Hassabis et al. 2007; Schacter and Addis 2007b; Schacter et al. 2017).

Equally important however, was the *absence* of any evidence linking executive dysfunction to the negative effects of acute alcohol consumption on EFT. In contrast to the EFT deficit that emerged following acute alcohol use, the capacity to complete all three executive function tasks was preserved. Additionally, only one of the three executive function measures were related to EFT in each condition, and the specific measure was not consistent across the two conditions. Regression analyses also showed that none of the executive function measures predicted EFT performance in the alcohol condition. Taken together, these data indicate that the effects of acute alcohol consumption on EFT are not underpinned by broader disturbances in executive function, or at least not the specific executive functions assessed in the present study, at the dose of alcohol administered. Given the noted importance of both dose and task complexity in considering the effects of acute alcohol consumption on cognitive performance, future research is now needed to establish whether the same or a different pattern of results emerges at higher concentrations of alcohol, or with different executive function tasks such as those that more heavily load specific executive operations (see e.g. Henry and Phillips 2006). In addition, given that EFT has been argued to involve a range of other cognitive abilities such as semantic memory (Irish 2016; Irish and Piguet 2013), and relational memory (Wiebels et al. 2020), future work should also consider how these might be impacted by acute alcohol use and potentially contribute to EFT impairment.

Finally, an exploratory component of this study was to examine sex effects in EFT following acute alcohol administration. Interestingly, but consistent with past research (Erol and Karpyak 2015; Fama et al. 2020; Mumenthaler et al. 1999), women attained a higher BrAC level than men even though they consumed an equivalent dose of alcohol adjusted for body weight. However, despite this difference, this did not translate to increased impairment in EFT performance in women in the alcohol group. Rather, women’s EFT performance in the alcohol group was comparable to men in that group, indicating that the level of alcohol-related impairment in cognition, at least in terms of EFT, does not appear to be related to sex.

In conclusion, these data provide the first empirical evidence that even a moderate dose of alcohol is sufficient to produce significant impairment in EFT and shows that these difficulties are related to the effects of acute alcohol consumption on episodic memory, but not executive functioning, and the effects did not vary as a function of biological sex. A potential limitation that should be noted, and which may temper conclusions regarding the functional relevance of executive functioning versus episodic memory in contributing to EFT is that the executive function measures were administered at a slightly lower BrAC (BrAC = 0.064) relative to when the episodic memory and EFT tests were administered (BrAC = 0.075). Steps should be taken in future designs to ensure equivalence here. The results of the present study could have important implications for individual decision making and the treatment of individuals with an alcohol use disorder, whilst also potentially furthering our understanding of why deleterious behaviours are so common under the influence of even moderate levels of alcohol. Further research is now required to gain a more nuanced understanding of exactly when and how EFT is compromised by acute alcohol use by assessing additional cognitive and neural mechanisms and at different levels of alcohol intoxication. Future research would also benefit from examining the effect of emotional valence on the ability to imagine the future under the influence of alcohol. Finally, although not the focus of the present study, future research should consider potential mechanisms that contribute to external responses (i.e. errors) following alcohol use, and in particular, the potential role of distraction or lack of focus that often accompanies alcohol consumption.

## Footnotes

1 It should be noted that definitions of social drinking vary across sociocultural contexts, and many different criteria have been proposed to define ‘levels’ of drinking, and not all of these would consider the criteria we have used here to qualify as social.

## References

[CR1] Addis DR (2018). Are episodic memories special? On the sameness of remembered and imagined event simulation. J R Soc N Z.

[CR2] Addis DR, Wong AT, Schacter DL (2008). Age-related changes in the episodic simulation of future events. Psychol Sci.

[CR3] Arbuthnott K, Frank J (2000). Trail making test, part B as a measure of executive control: validation using a set-switching paradigm. J Clin Exp Neuropsychol.

[CR4] Atance CM, O’Neill DK (2001) Episodic future thinking. Trends Cogn Sci 5:533–539. 10.1016/S1364-6613(00)01804-0 10.1016/s1364-6613(00)01804-0 11728911

[CR5] Baddeley A, Emslie H, Nimmo-Smith I (1993). The spot-the-word test: a robust estimate of verbal intelligence based on lexical decision. Br J Clin Psychol.

[CR6] Bradley MM, Lang PJ (1999) Affective norms for English words (ANEW): instruction manual and affective ratings

[CR7] Buckner RL, Carroll DC (2007). Self-projection and the brain. Trends Cogn Sci.

[CR8] Burgess PW, Shallice T (1997). The Hayling and Brixton tests.

[CR9] Charness ME (1990) Alcohol and the brain alcohol health and research world. 14:85–90

[CR10] Cohen J (1988) Statistical power analysis for the behavioral sciences. 2nd edn. Erlbaum, Hillsdale, NJ

[CR11] Cole SN, Morrison CM, Conway MA (2013). Episodic future thinking: linking neuropsychological performance with episodic detail in young and old adults. Q J Exp Psychol.

[CR12] Curran HV, Weingartner H, Baddeley A, Kopelman M, Wilson B (2002). Psychopharmacology of human memory. Handbook of memory disorders.

[CR13] D'Argembeau A, Raffard S, Van der Linden M (2008). Remembering the past and imagining the future in schizophrenia. J Abnorm Psychol.

[CR14] Day AM, Kahler CW, Ahern DC, Clark US (2015). Executive functioning in alcohol use studies: a brief review of findings and challenges in assessment. Curr Drug Abuse Rev.

[CR15] Eckardt MJ (1998). Effects of moderate alcohol consumption on the central nervous system alcoholism. Clin Exp Res.

[CR16] Erol A, Karpyak VM (2015). Sex and gender-related differences in alcohol use and its consequences: contemporary knowledge and future research considerations. Drug Alcohol Depend.

[CR17] Fama R, Le Berre A-P, Sullivan EV (2020) Alcohol’s unique effects on cognition in women: a 2020 (re) view to envision future research and treatment alcohol research. Curr Rev. 10.35946/arcr.v40.2.03 10.35946/arcr.v40.2.03 PMC7473713 32923307

[CR18] Field M, Wiers RW, Christiansen P, Fillmore MT, Verster JC (2010). Acute alcohol effects on inhibitory control and implicit cognition: implications for loss of control over drinking. Alcohol Clin Exp Res.

[CR19] Fillmore MT (2007). Acute alcohol-induced impairment of cognitive functions: past and present findings. Int J Disabil Hum Dev.

[CR20] Gott C, Lah S (2014). Episodic future thinking in children compared to adolescents. Child Neuropsychol.

[CR21] Grant S, Contoreggi C, London ED (2000). Drug abusers show impaired performance in a laboratory test of decision making. Neuropsychologia.

[CR22] Griffiths A, Hill R, Morgan C, Rendell PG, Karimi K, Wanagaratne S, Curran HV (2012) Prospective memory and future event simulation in individuals with alcohol dependence Addiction 107:1809–1816. 10.1111/j.1360-0443.2012.03941.x 10.1111/j.1360-0443.2012.03941.x 22578026

[CR23] Hassabis D, Kumaran D, Vann SD, Maguire EA (2007). Patients with hippocampal amnesia cannot imagine new experiences. Proc Natl Acad Sci.

[CR24] Henry JD, Phillips LH (2006). Covariates of production and perseveration on tests of phonemic, semantic and alternating fluency in normal aging. Aging Neuropsychol Cogn.

[CR25] Holloway FA (1994). Low-dose alcohol effects on human behavior and performance: a review of post-1984 research. Office of Aviation Medicine, Washington, DC.

[CR26] Hull JG, Bond CF (1986). Social and behavioral consequences of alcohol consumption and expectancy: a meta-analysis. Psychol Bull.

[CR27] Irish M, Kourken M, Klein SB, Szpunar KK (2016). Semantic memory as the essential scaffold for future-oriented mental time travel. Seeing the future: Theoretical perspectives on future-oriented mental time travel.

[CR28] Irish M, Piguet O (2013). The pivotal role of semantic memory in remembering the past and imagining the future. Front Behav Neurosci.

[CR29] Irish M, Lawlor BA, O'Mara SM, Coen RF (2011). Impaired capacity for autonoetic reliving during autobiographical event recall in mild Alzheimer’s disease. Cortex.

[CR30] Jacob A, Wang P (2020). Alcohol intoxication and cognition: implications on mechanisms and therapeutic strategies. Front Neurosci.

[CR31] Leitz JR, Morgan CJ, Bisby JA, Rendell PG, Curran HV (2009). Global impairment of prospective memory following acute alcohol. Psychopharmacology.

[CR32] Lind SE, Bowler DM (2010). Episodic memory and episodic future thinking in adults with autism. J Abnorm Psychol.

[CR33] Mehrabian A, Russell JA (1978). A questionnaire measure of habitual alcohol use. Psychol Rep.

[CR34] Mercuri K, Terrett G, Henry JD, Bailey PE, Curran HV, Rendell PG (2015). Episodic foresight deficits in long-term opiate users. Psychopharmacology.

[CR35] Mercuri K, Terrett G, Henry JD, Curran HV, Elliott M, Rendell PG (2018). Episodic foresight deficits in regular, but not recreational, cannabis users. J Psychopharmacol.

[CR36] Mintzer MΖ (2007). The acute effects of alcohol on memory: a review of laboratory studies in healthy adults. Int J Disabil Hum Dev.

[CR37] Mumenthaler MS, Taylor JL, O’Hara R, Yesavage JA (1999). Gender differences in moderate drinking effects. Alcohol Res Health.

[CR38] Oscar-Berman M, Marinković K (2007). Alcohol: effects on neurobehavioral functions and the brain. Neuropsychol Rev.

[CR39] Paraskevaides T, Morgan CJ, Leitz JR, Bisby JA, Rendell PG, Curran HV (2010). Drinking and future thinking: acute effects of alcohol on prospective memory and future simulation. Psychopharmacology.

[CR40] Roberts RP, Schacter DL, Addis DR (2018). Scene construction and relational processing: separable constructs?. Cereb Cortex.

[CR41] Ryback RS (1971). The continuum and specificity of the effects of alcohol on memory: a review. Q J Stud Alcohol.

[CR42] Schacter DL, Addis DR (2007). The cognitive neuroscience of constructive memory: remembering the past and imagining the future. Philos Trans R Soc B Biol Sci.

[CR43] Schacter DL, Addis DR (2007). On the constructive episodic simulation of past and future events. Behav Brain Sci.

[CR44] Schacter DL, Addis DR, Buckner RL (2007). Remembering the past to imagine the future: the prospective brain. Nat Rev Neurosci.

[CR45] Schacter DL, Addis DR, Buckner RL (2008). Episodic simulation of future events: concepts, data, and applications. Ann N Y Acad Sci.

[CR46] Schacter DL, Benoit RG, Szpunar KK (2017). Episodic future thinking: mechanisms and functions. Curr Opin Behav Sci.

[CR47] Squire LR, Stark CE, Clark RE (2004). The medial temporal lobe. Annu Rev Neurosci.

[CR48] Strauss E, Sherman EM, Spreen O (2006). A compendium of neuropsychological tests: administration, norms, and commentary.

[CR49] Suddendorf T (2010). Episodic memory versus episodic foresight: similarities and differences Wiley Interdisciplinary Reviews. Cogn Sci.

[CR50] Suddendorf T, Corballis MC (2007). The evolution of foresight: what is mental time travel, and is it unique to humans?. Behav Brain Sci.

[CR51] Suddendorf T, Henry J (2013). Proximate and ultimate perspectives on memory. J Appl Res Mem Cogn.

[CR52] Suddendorf T, Addis DR, Corballis MC (2009). Mental time travel and shaping of the human mind. Philos Trans R Soc B Biol Sci.

[CR53] Szpunar KK (2010). Episodic future thought: an emerging concept. Perspect Psychol Sci.

[CR54] Tabachnick B, Fidell L (2014). Using multivariate statistics.

[CR55] Tombaugh TN, Kozak J, Rees L (1999). Normative data stratified by age and education for two measures of verbal fluency: FAS and animal naming. Arch Clin Neuropsychol.

[CR56] Townshend J, Duka T (2002). Patterns of alcohol drinking in a population of young social drinkers: a comparison of questionnaire and diary measures. Alcohol Alcohol.

[CR57] Van Skike CE, Goodlett C, Matthews DB (2019). Acute alcohol and cognition: remembering what it causes us to forget. Alcohol.

[CR58] Weissenborn R, Duka T (2003). Acute alcohol effects on cognitive function in social drinkers: their relationship to drinking habits. Psychopharmacology.

[CR59] White AM (2003). What happened? Alcohol, memory blackouts, and the brain. Alcohol Res Health.

[CR60] Wiebels K, Addis DR, Moreau D, van Mulukom V, Onderdijk KE, Roberts RP (2020) Relational processing demands and the role of spatial context in the construction of episodic simulations. J Exp Psychol Learn Mem Cogn 1–18. 10.1037/xlm0000831 10.1037/xlm0000831 32134319

[CR61] Zigmond AS, Snaith RP (1983). The hospital anxiety and depression scale. Acta Psychiatr Scand.

